# Bioinspired supramolecular nanosheets of zinc chlorophyll assemblies

**DOI:** 10.1038/s41598-019-50026-1

**Published:** 2019-10-02

**Authors:** Sunao Shoji, Tetsuya Ogawa, Shogo Matsubara, Hitoshi Tamiaki

**Affiliations:** 1 Graduate School of Life Sciences, Ritsumeikan University, Kusatsu, Shiga, 525-8577 Japan; 2 Institute for Chemical Research, Kyoto University, Uji, Kyoto, 611-0011 Japan; 3 Present Address: Division of Applied Chemistry, Faculty of Engineering, Hokkaido University, Sapporo, Hokkaido, 060-8628 Japan

**Keywords:** Antenna complex, Self-assembly

## Abstract

Two-dimensional sheet-like supramolecules have attracted much attention from the viewpoints of their potential application as functional (nano)materials due to unique physical and chemical properties. One of the supramolecular sheet-like nanostructures in nature is visible in the self-assemblies of bacteriochlorophyll-*c*–*f* pigments inside chlorosomes, which are major components in the antenna systems of photosynthetic green bacteria. Herein, we report artificial chlorosomal supramolecular nanosheets prepared by the self-assembly of a synthetic zinc 3^1^-methoxy-chlorophyll derivative having amide and urea groups in the substituent at the 17-position. The semi-synthetic zinc chlorophyll derivative kinetically formed dimeric species and transformed into more thermodynamically stable chlorosomal *J*-aggregates in the solid state. The kinetically and thermodynamically formed self-assemblies had particle-like and sheet-like supramolecular nanostructures, respectively. The resulting nanosheets of biomimetic chlorosomal *J*-aggregates had flat surfaces and well-ordered supramolecular structures. The artificial sheet-like nanomaterial mimicking chlorosomal bacteriochlorophyll-*c*–*f J*-aggregates was first constructed by the model molecule, and is potentially useful for various applications including artificial light-harvesting antennas and photosyntheses.

## Introduction

**Figure 1:**
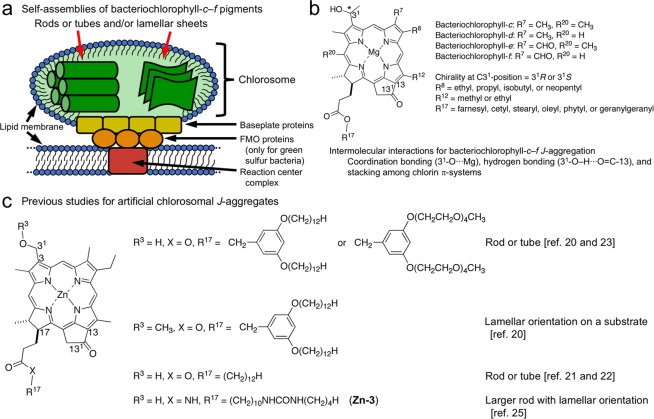
Natural and artificial chlorosomal systems. (**a**) Schematic of photosynthetic apparatuses in photosynthetic green bacteria. (**b**) Molecular structures of bacteriochlorophyll-*c*–*f* molecules. (**c**) Synthetic chlorophyll derivatives reported as models of bacteriochlorophyll-*d*.

Two-dimensional sheet-like nanostructures have been made by organic and/or inorganic materials, for example, graphene, supramolecules, polymers, covalent organic frameworks, metal organic frameworks, and hexagonal boron nitride, and interested in wide fields such as electronics, optoelectronics, catalysts, energy storage, energy generation, sensors, separation, and biomedicines due to their unique physical and chemical properties^1–3^. Supramolecular assembling systems are progressive strategy for preparing well-ordered nanostructures^4–6^. In nature, one of the supramolecular sheet-like nanostructures have been observed in major light-harvesting antenna systems of photosynthetic green bacteria, called chlorosomes^7–9^. Chlorosomes are egg-like, ellipsoidal, extramembranous antenna apparatuses (Fig. 1a), and one of the most efficient photofunctional nanodevices in nature, which have attracted attention from the viewpoints of their functions with photophysical properties, supramolecular structures, and nanostructures^10–13^. A single chlorosome contains a large amount of bacteriochlorophyll-*c*, *d*, *e*, and *f* pigments (Fig. 1b), magnesium complexes of 3^1^-hydroxy-13^1^-oxo-chlorin, which are surrounded by lipid monolayers and form *J*-aggregates in hydrophobic environments without any protein assistance^14,15^. Their self-assembly is mainly organized by coordination bonding (3^1^-O∙∙∙Mg), hydrogen bonding (3^1^-O–H∙∙∙O=C-13), and π–π stacking of chlorin skeletons^16,17^. Their supramolecular (nano)structures are still in discussion, but suggested to be rods, tubes, and/or lamellar sheets.

Model compounds of such chlorosomal bacteriochlorophyll-*c*–*f* pigments have been synthesized from naturally occurring chlorophyll-*a*^18,19^. Their rod-, tube-, and/or lamellar-shaped supramolecular nanostructures have been prepared using model molecules in solution and solid states as well as on substrates (Fig. 1c)^20–24^. Recently, we have reported that a zinc 3^1^-hydroxy-13^1^-oxo-chlorin having amide and urea groups in the 17-substituent self-assembled to form rod-like supramolecular nanostructures with a spacing of 1.4 nm^25^. In this study, we synthesized zinc 3^1^-methoxy-chlorophyll derivatives lacking the 3^1^-hydroxy group, which was requisite for chlorosomal *J*-aggregation through specific hydrogen bonding (3^1^-O–H∙∙∙O=C-13). The zinc 3^1^-methoxy-13^1^-oxo-chlorin having amide and urea groups in the 17-substituent gradually formed chlorosomal *J*-aggregates in the solid state, and the supramolecular nanostructures of the resulting solids were mainly nanosheets that mimic the natural bacteriochlorophyll-*c*–*f* supramolecules in chlorosomes. This is the first report of biomimetic supramolecular nanosheets of chlorosomal *J*-aggregates being prepared from a synthetic zinc chlorophyll derivative.

## Results and Discussion

**Figure 2:**
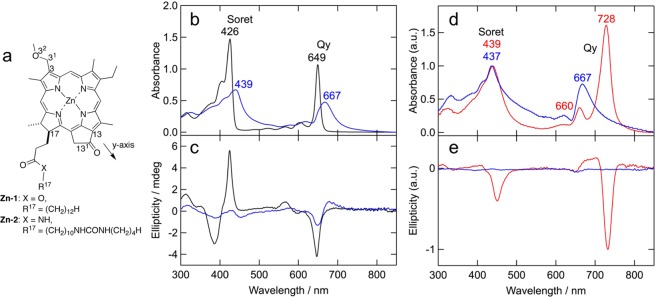
Supramolecular assembly of zinc 3^1^-methoxy-chlorophyll derivatives. (**a**) Molecular structures of **Zn-1** and **Zn-2**. (**b**) UV-Vis-NIR absorption and (**c**) CD spectra of **Zn-2** (10 μM) in THF (black), and THF/hexane (1:99, vol/vol) (blue). (**d**) UV-Vis-NIR absorption and (**e**) CD spectra of **Zn-2** solids prepared from THF/hexane (5:95, vol/vol) (100 μM) just after preparation (blue) and after standing in solution for 1 week (red).

Zinc chlorophyll derivatives **Zn-1** and **Zn-2** (Fig. 2a) were synthesized from methyl 3-devinyl-3-methoxymethyl-pyropheophorbide-*a*, which was prepared from naturally occurring chlorophyll-*a* extracted from commercially available Spirulina powders according to reported procedures (see synthetic details and spectral data in Supporting Information). The **Zn-1** and **Zn-2** molecules have ester and amide/urea groups, respectively, in the substituents at the 17-position, which are designed as without and with hydrogen bonding moieties. The UV-Vis absorption spectra of **Zn-1** and **Zn-2** in tetrahydrofuran (THF) at a concentration of 10 μM showed their monomeric states with Qy/Soret maxima at 649/426 nm (Fig. 2b, black line). The self-assembly of the zinc 3^1^-methoxy-chlorins in a hydrophobic hexane-based solution was examined. A solution of the zinc chlorin in THF was added to 99-times the volume of hexane (total concentration: 10 μM). The UV-Vis-NIR absorption spectrum of **Zn-2** exhibited red-shifted Qy/Soret maxima at 667/439 nm (Fig. 2b, blue line), which are ascribable to dimeric species, while **Zn-1** gave only a monomeric state similar to its THF solution. Additionally, excitonically coupled circular dichroism (CD) signals were observed in the Qy region for the hexane-based solution of **Zn-2** (Fig. 2c, blue), indicating that the molecular dipole moments of the chlorin π-systems were aligned along the y-axis. These results indicate that **Zn-2** primarily form the dimeric species by intermolecular hydrogen bonding of its amide and urea groups in the 17-substituent, which readily induce stacking of the chlorin π-systems as well as coordination of the 3^1^-O and central zinc in a dimer.

At a 10-fold higher concentration (100 μM) in THF/hexane (5:95, vol/vol), **Zn-2** was precipitated immediately, while **Zn-1** was dissolved in the solution as the monomeric state (Fig. S1). The resulting **Zn-2** solids just after preparation were adsorbed on a quartz substrate. The UV-Vis-NIR absorption spectrum exhibited Qy/Soret maxima at 667/437 nm (Fig. 2d, blue line), which was similar to that diluted in the hexane-based solution (Fig. 2b, blue line). After standing the precipitates in the above mixed solvent for 1 week in the dark at room temperature, the color of the **Zn-2** solids was changed to dark green from blue-green, while **Zn-1** was still dissolved in the mixed solvent to give no color change (Fig. S1). The resulting dark green solids were adsorbed onto a quartz substrate. The UV-Vis-NIR absorption spectrum showed further red-shifted Qy/Soret maxima at 728/439 nm (Fig. 2d, red line), and the excitonically coupled CD signals in the Qy region were enhanced (Fig. 2e, red line). These spectroscopic data indicate that the **Zn-2** solids were gradually transformed from dimeric or small oligomeric species into chlorosomal *J*-aggregates, in which the molecular dipole moments of the **Zn-2** molecules were arranged along the y-axis in a well-ordered manner. Thus, **Zn-2** molecules form dimeric species kinetically and transform into more thermodynamically stable *J*-aggregates in the solid states. The resulting **Zn-2** chlorosomal *J*-aggregates on a quartz substrate exhibited a 752-nm fluorescence emission peak at the excitation of the Soret band (Fig. S2).

**Figure 3:**
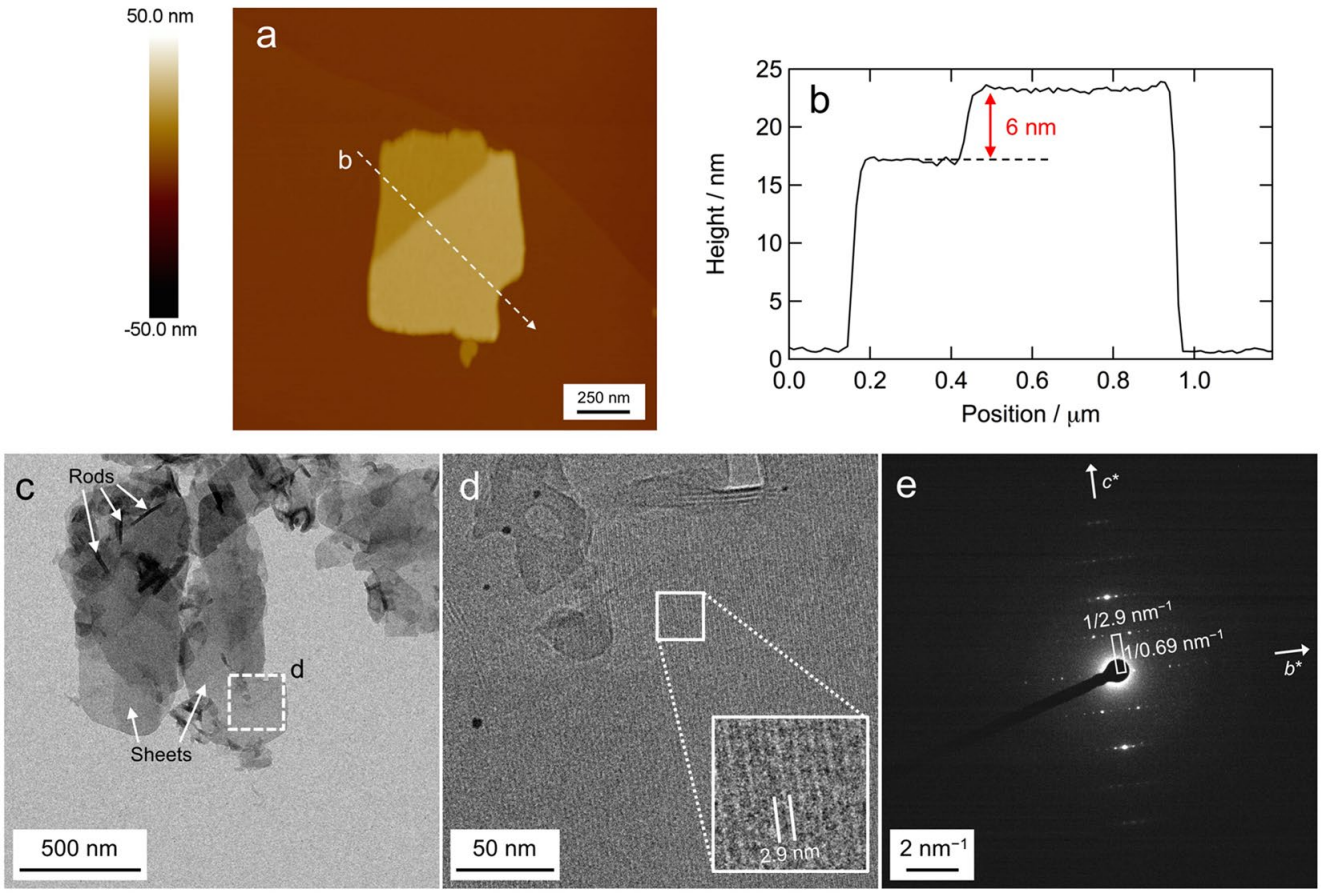
Supramolecular nanosheets of **Zn-2** self-assemblies formed upon standing in THF/hexane (5:95, vol/vol) for one week. Tapping-mode AFM analysis of **Zn-2** *J*-aggregates on an HOPG substrate, (**a**) AFM height image and (**b**) cross-section analysis. Cryo-TEM analysis of **Zn-2** on a carbon-coated copper grid, (**c**) and (**d**) cryo-TEM images and (**e**) electron beam diffraction.

**Figure 4:**
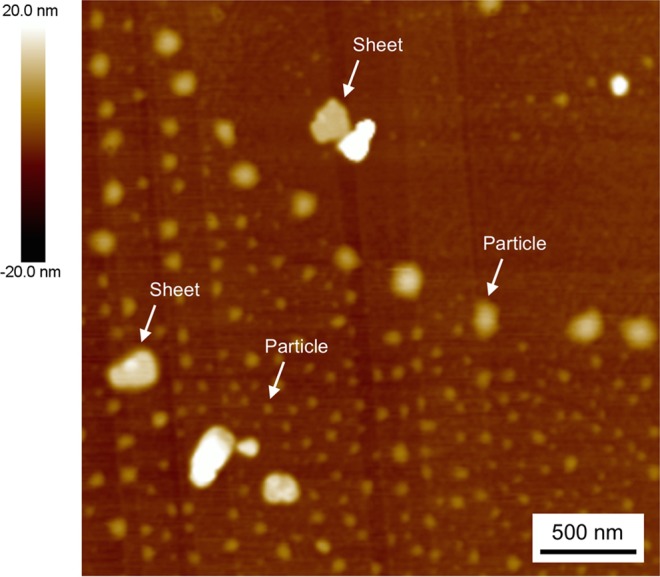
A tapping mode AFM height image of **Zn-2** just after preparation in THF/hexane (5:95, vol/vol, 100 μM); the sample was dispersed by ultrasonication and drop-cast on an HOPG substrate.

The **Zn-2** solids produced in THF/hexane (5:95, vol/vol) after standing for one week were dispersed by ultrasonication and the resulting suspension was drop-cast onto a substrate to analyze their supramolecular nanostructures using tapping-mode atomic force microscopy (AFM). The AFM height image of the **Zn-2** self-assemblies on a highly oriented pyrolytic graphite (HOPG) substrate showed sheet-like supramolecular nanostructures with heights of 15–25 nm (Figs. 3a and S3a). The cross-sectional analysis showed that the surface of the nanosheet was flat over a submicrometer range (Figs. 3b and S3b–e). The nanosheet had a step of 6 nm (Fig. 3b), suggesting a cleavage of a single layer of the nanosheet. The **Zn-2** solids produced in THF/hexane (5:95, vol/vol) just after preparation were also examined using tapping-mode AFM. The AFM images showed mainly particle-like nanostructures with heights of 1–25 nm and partially nanosheets with heights of 13–21 nm (Fig. 4). Thus, the results indicate that nanosheets of **Zn-2** *J*-aggregates are more thermodynamically stable nanostructures and that they are gradually formed from amorphous particle-like aggregates of kinetically favored dimeric species.

The aforementioned suspension of **Zn-2** *J*-aggregates was similarly drop-cast onto a carbon-coated copper grid, and analyzed using cryo-transmission electron microscopy (cryo-TEM). Cryo-TEM images also mainly showed supramolecular nanosheets (Fig. 3c) similarly as in AFM analysis (rod-like nanostructures were partially visible in the cryo-TEM images of **Zn-2** *J*-aggregates in Figs. 3c and S4). Moreover, black stripes with a spacing of 2.9 nm were visible in the magnified cryo-TEM image (Figs. 3d and S5). Taking a closer look at this image, it can be seen that one black stripe consists of a pair of black lines (Fig. 3d, inset). Since the cryo-TEM image was obtained without stain, the black lines in the Fig. 3d image were based on the zinc atoms of **Zn-2** molecules. The electron beam diffraction of the nanosheet showed an orthogonal pattern (Fig. 3e). Assuming an orthorhombic unit cell (*a* = 6 nm, *b* = 2.9 nm, *c* = 0.69 nm) from Fig. 3e as well as Fig. 3b, there is a systematic absence of *00 l* (*l*: odd) reflections by extinction rule. This indicates the presence of the 2_1_ screw axis along the *c*-axis. The 0.69-nm distance between adjacent zinc chlorophyll molecules was similar to previously reported results for microcrystalline of a cadmium BChl-*d* analog^26^ as well as self-assemblies of **Zn-3**^25^ (Fig. 1c, bottom), the 3^1^-hydroxylated analog of **Zn-2**. **Zn-3** formed a large rod-shaped supramolecular nanostructure through intermolecular hydrogen bonding between 3^1^-OH and 13-C=O, and its striped orientation was parallel to the long axis of the rod. In the present study, 3^1^-methoxylated **Zn-2** forms no hydrogen bonding between 3^1^-OCH_3_ and 13-C=O and also weakens the coordination ability of 3^1^-O to central zinc due to the sterically bulky 3^2^-methyl group, inducing less chlorosomal *J*-aggregation and relatively enhancing the intermolecular hydrogen bonding of the amide and urea groups in the 17-substituents. The balance of intermolecular interactions is the driving force for the slow growth of two-dimensional **Zn-2** *J*-aggregates as major supramolecular nanostructures under the present conditions.

**Figure 5:**
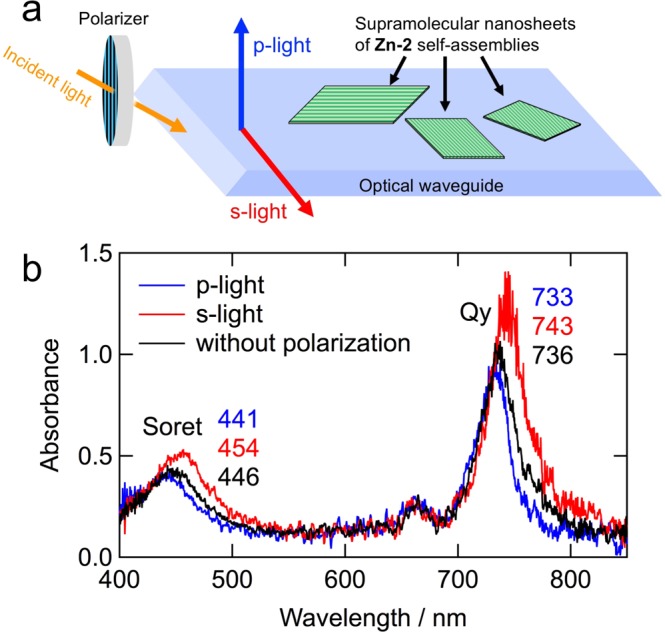
Vis-NIR absorption spectra of **Zn-2** nanosheets using optical waveguide. (**a**) Schematic of the measurement and (**b**) Vis-NIR absorption spectra of **Zn-2** nanosheets on an optical waveguide using p- (blue) and s-light (red) as well as non-polarized light (black).

The two-dimensional **Zn-2** nanosheets were investigated in terms of their anisotropy to further elucidate their supramolecular structure. The solid **Zn-2** nanosheets were drop-cast onto a quartz optical waveguide and their Vis-NIR absorption spectra were measured using polarized light, perpendicular (p-light) and parallel (s-light) to the surface (Fig. 5a). The Vis-NIR absorption spectrum of the nanosheet without polarization showed Qy/Soret maxima at 736/446 nm (Fig. 5b, black line), which was similar to those measured using conventional transmission mode (Fig. 2d, red line). The p- and s-light absorption spectra showed Qy/Soret maxima at 733/441 and 743/454 nm, respectively (Fig. 5b, blue and red lines). These observations revealed that the molecular dipole moments of **Zn-2** molecules along the y-axis were arranged in two situations with both vertical and horizontal fashions. The Qy and Soret absorption peaks of the nanosheets under s-light were observed at longer wavelength regions than those under p-light, indicating that **Zn-2** molecules stacked more strongly in parallel direction to the optical waveguide surface than that in perpendicular one. This suggests that **Zn-2** molecules form linearly stepped oligomers favorably orientated parallel to the quartz surface. It is consistent with the microscopic analyses, which reveal a striped orientation in the nanosheet parallel to the surface of the substrate. Thus, a single striped layer is ascribable to the linearly stepped oligomeric **Zn-2** molecules.

**Figure 6:**
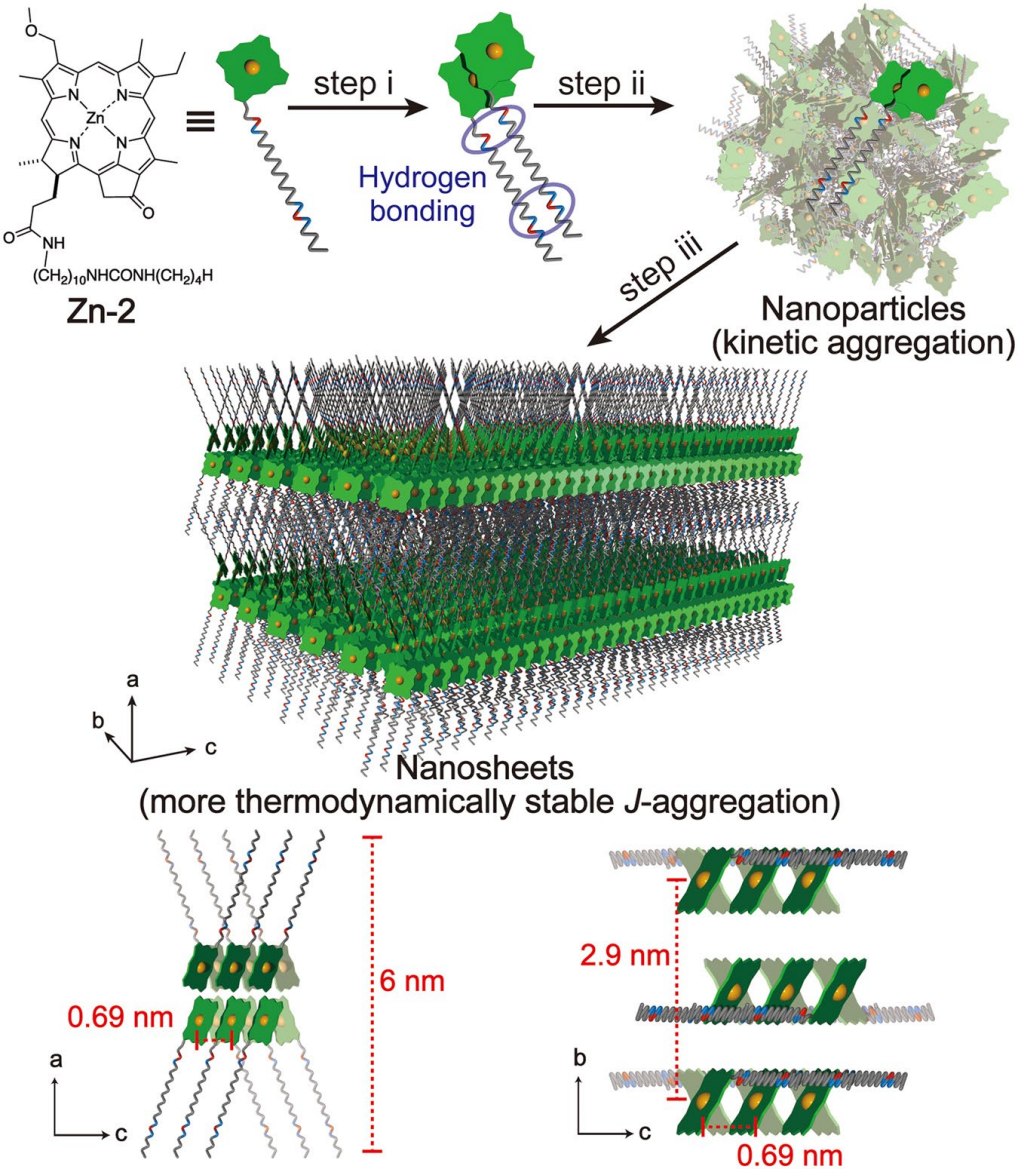
Schematic drawing of the **Zn-2** chlorosomal *J*-aggregation process.

The self-assembly of **Zn-2** molecules is schematically represented in Fig. 6. The **Zn-2** molecules kinetically formed a dimeric species based on its intermolecular hydrogen bonding of amide and urea groups in a hydrophobic environment (Fig. 6, step i). The dimeric species immediately formed nanoparticles (Fig. 6, step ii)^23,24^, which gradually transformed into the more thermodynamically stable supramolecular nanosheets of chlorosomal *J*-aggregates (Fig. 6, step iii). Appropriate crystal structure model of **Zn-2** molecules in nanosheets consistent with the cryo-TEM data and the extinction rule is shown in middle and bottom of Fig. 6. Two arrays of zinc atoms along *c*-axis in *bc*-plane (*a*-axis projection) corresponds to the black lines in cryo-TEM image (Fig. 3d). It is noted that the supramolecular structures of bacteriochlorophyll-*c*–*f* assemblies are still in discussion^23,24,27–29^, and the supramolecular structure of **Zn-2** molecules in the nanosheet and the ligation of the central zinc (α or β) as well as the configuration between the 3^1^-methoxy moiety and 17-methylene group [*syn* (same side) and *anti* (opposite side)] are unclear. The **Zn-2** supramolecular nanosheets had a striped pattern, which is reminiscent of a lamellar sheet nanostructure of natural chlorosomal bacteriochlorophyll assemblies. A natural chlorosome consists of epimeric mixtures of bacteriochlorophyll-*c*–*f* homologs and a variety of carotenoids, quinones, and waxes, which are surrounded by a lipid monolayer. Although all these components should affect their supramolecular nanostructures of bacteriochlorophyll-*c*–*f* assemblies, their essential supramolecular (nano)structures are controlled by themselves. For the present **Zn-2** *J*-aggregates, supramolecular nanosheets are simply constructed by intermolecular interactions of coordination (3^1^-O∙∙∙Zn) and hydrogen bonding (C=O∙∙∙H–N) as well as π–π stacking of the chlorins in the solid state.

## Conclusion

Zinc 3^1^-methoxy-chlorophyll derivatives **Zn-1** and **Zn-2** were synthesized from naturally occurring chlorophyll-*a*. **Zn-2** having amide and urea groups in the 17-substituent kinetically formed a dimeric species that gradually transformed into the more thermodynamically stable chlorosomal *J*-aggregates in the solid state, while **Zn-1** bearing a dodecyl at the 17-propionate residue formed neither dimers nor large aggregates. The dimerization and *J*-aggregation of **Zn-2** molecules are driven by its intermolecular hydrogen bonding of amide and urea moieties in the 17-substituents as well as the coordination (3^1^-O∙∙∙Zn) and π–π stacking of chlorin π-systems. The structural transformation of the chlorophyll supramolecules was first observed in the solid state using spectroscopy. The major supramolecular nanostructures of the more thermodynamically stable **Zn-2** *J*-aggregates were nanosheets, which were first constructed. The present two-dimensional nanomaterial photophysically and nanostructurally mimics chlorosomal bacteriochlorophyll-*c*–*f J*-aggregates. The supramolecular nanosheets (the present study) and nanotubes^20–22^, which are reminiscent of covalently-bonded graphene and carbon nanotube, respectively, could be prepared using zinc chlorophyll derivatives. The bioinspired supramolecular nanosheets of chlorosomal *J*-aggregates have various potential applications, such as artificial light-harvesting antennas and photosynthesis, dye-sensitized solar cells, catalytic reactions, and photodynamic therapy.

## Methods

### Samples

Chlorophyll-*a* was extracted from commercially available cyanobacterial spirulina powders (DIC LIFETEC). Synthetic procedures and spectral data for **Zn-1** and **Zn-2** are described in Supplementary Information. The samples in a solution at 10 μM for UV-Vis-NIR absorption measurements were prepared as follows. The samples were weighed on Ultra-Microbalance XPR2UV (Mettler Toledo), and dissolved in THF, then an aliquot of the solution was dried in vacuo. The residue (20 nmol) was dissolved in only THF (2 mL), or first dissolved in THF (0.02 mL) that was then added to hexane (1.98 mL) in a 1-cm quartz cuvette. The samples at 100 μM were prepared as follows. The samples (1 μmol) were first dissolved in THF (0.5 mL) and diluted with hexane (9.5 mL). The resulting suspension of **Zn-2** in THF/hexane (5:95, vol/vol) was stored at room temperature in the dark for one week to prepare two-dimensional nanosheets. The solid samples of **Zn-2** in THF/hexane (5:95, vol/vol) were dispersed using ultrasonication, and a quartz plate was dipped in this suspension and then dried more than 10 times to adsorb self-assemblies of **Zn-2** onto the quartz surface for UV-Vis-NIR absorption measurements performed using conventional transmission mode. The **Zn-2** solids were drop-cast onto a quartz optical waveguide (System Instrument) for the measurement of Vis-NIR absorption spectra in total reflection mode in air. For AFM measurements, the solid samples of **Zn-2** in the above hexane-based solution were dispersed by ultrasonication for 30 seconds and the resulting suspensions were drop-cast onto HOPG substrates (Bruker) and dried immediately by purging nitrogen gas. For the cryo-TEM measurements, a suspension of **Zn-2** was drop-cast onto a carbon coated copper grid. The solvents used for the optical and microscopic measurements were purchased from Nacalai Tesque as reagents prepared specifically for spectroscopic experiments.

### UV-Vis-NIR absorption spectroscopy

UV-Vis-NIR absorption spectra in transmission mode were measured at room temperature on a Hitachi U-3500 spectrophotometer. Vis-NIR absorption spectra in a total reflection mode using an optical waveguide were measured at room temperature with a System Instruments SIS-50 spectrophotometer.

### CD spectroscopy

CD spectra were measured at room temperature on a Jasco J-720W spectropolarimeter.

### AFM

AFM was performed with a Bruker Multimode 8 system in tapping mode in air. An HOPG substrate was freshly cleaved before drop-casting the samples. A silicon cantilever (Bruker, MPP-11100–10) was used.

### Cryo-TEM

Cryo-TEM was performed with a JEOL JEM-2100F (G5) microscope operated at the acceleration voltage of 200 kV and measured at liquid helium temperature (4.2 K).

### Open access

This article is licensed under a Creative Commons Attribution 4.0 International License, which permits use, sharing, adaptation, distribution and reproduction in any medium or format, as long as you give appropriate credit to the original author(s) and the source, provide a link to the Creative Commons license, and indicate if changes were made. The images or other third party material in this article are included in the article’s Creative Commons license, unless indicated otherwise in a credit line to the material. If material is not included in the article’s Creative Commons license and your intended use is not permitted by statutory regulation or exceeds the permitted use, you will need to obtain permission directly from the copyright holder. To view a copy of this license, visit http://creativecommons.org/licenses/by/4.0/.

## Supplementary information

Supplementary Information

## Data Availability

The data that support the findings of this study are available within the article and its Supplementary Information files.
