# Supplementary material for: Bioinspired supramolecular nanosheets of zinc chlorophyll assemblies
Source: Sci Rep. 2019 Oct 2;9:14006. doi: 10.1038/s41598-019-50026-1 (PMC6773735; doi:10.1038/s41598-019-50026-1)
Supplement: Supplementary file 1 — Supplementary Information [file 41598_2019_50026_MOESM1_ESM.doc]

**Supplementary Information**

**Bioinspired supramolecular nanosheets of zinc chlorophyll assemblies**

Sunao Shoji,^1,†^ Tetsuya Ogawa,^2^ Shogo Matsubara,^1^ Hitoshi Tamiaki^1^

^1^Graduate School of Life Sciences, Ritsumeikan University, Kusatsu, Shiga 525-8577, Japan, ^2^Institute for Chemical Research, Kyoto University, Uji, Kyoto 611-0011, Japan

^†^Present address: Division of Applied Chemistry, Faculty of Engineering, Hokkaido University, Sapporo, Hokkaido 060-8628, Japan

Correspondence and requests for materials should be addressed to S.S. (email: s-shoji@eng.hokudai.ac.jp) or H.T. (email: tamiaki@fc.ritsumei.ac.jp)

**1. Experimental**

UV-Vis-NIR absorption, AFM, and cryo-TEM data were measured with an apparatus described in the main text. The fluorescence emission spectra were obtained using a Hamamatsu Photonics C9920-03G spectrometer. ^1^H NMR spectra were recorded at 293 K on a JEOL ECA-600 (600 MHz) spectrometer; Si(CH_3_)_4_ (*δ*_H_ = 0.00 ppm) was used as an internal reference. High-resolution (HR) MS data were recorded on a Bruker micrOTOF II spectrometer equipped with atmospheric pressure chemical ionization (APCI) probe in positive mode in a MeOH solution. Agilent Technologies APCI-L low concentration tuning mix was used as an internal reference.

**2. Synthesis of zinc 3^1^-methoxy-chlorophyll derivatives**


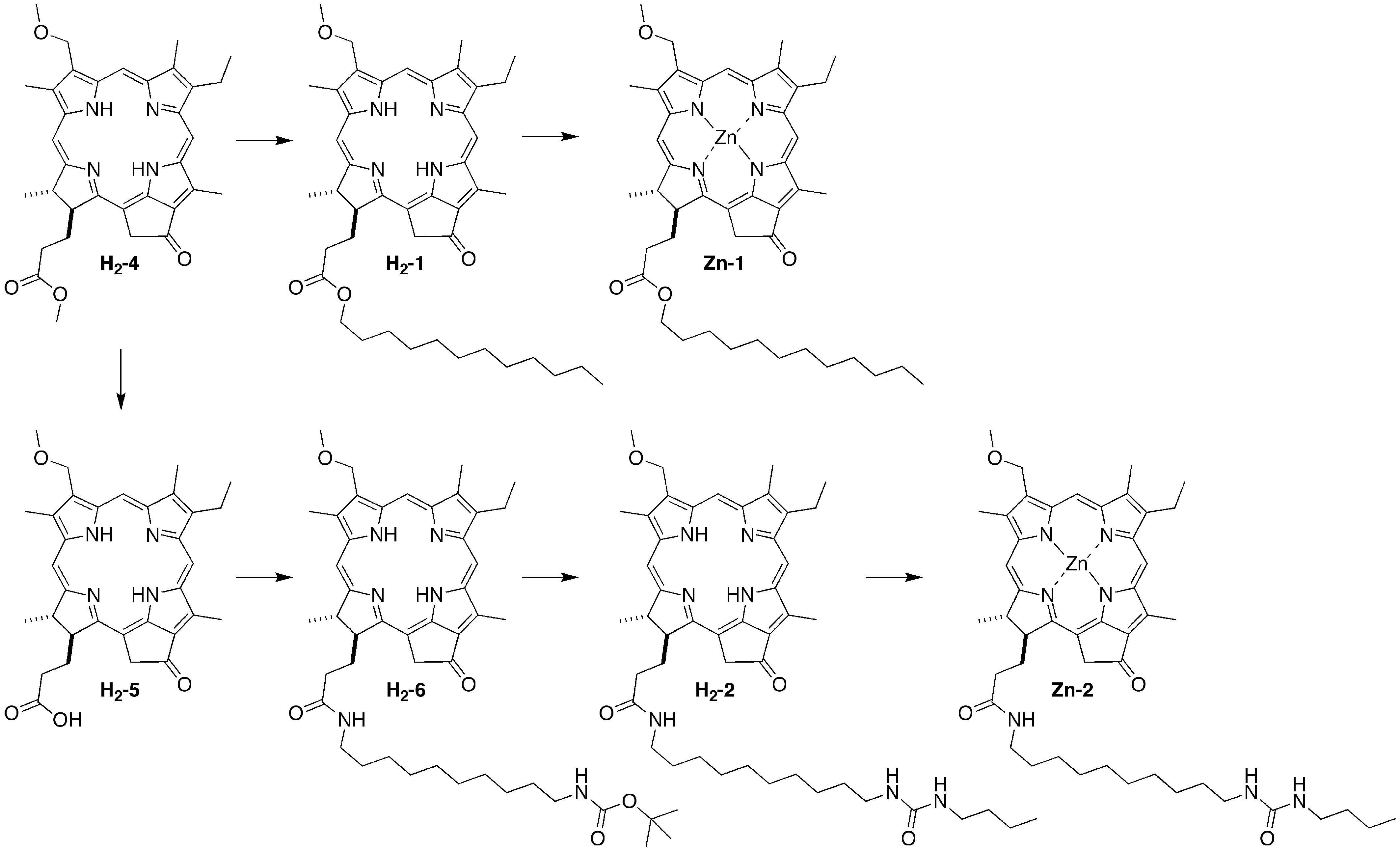


**Scheme S1.** Synthetic scheme of **Zn-1** and **Zn-2**.

3-Devinyl-3-methoxymethyl-pyropheophorbide-*a* methyl ester (**H_2_-4**)

The titled compound was synthesized from naturally occurring chlorophyll-*a* according to the reported procedures (see Supplementary references 1–4).

3-Devinyl-3-methoxymethyl-pyropheophorbide-*a* (**H_2_-5**)

The titled compound was synthesized according to the reported procedures (see Supplementary references 3 and 4).

3-Devinyl-methoxymethyl-pyropheophorbide-*a* dodecyl ester (**H_2_-1**)

Methyl ester **H_2_-4** (29.6 mg, 52.2 μmol), 1-dodecanol (197.5 mg, 1.06 mmol), and bis(dibutylchlorotin(IV)) oxide (11.2 mg, 20.3 μmol) were dissolved in toluene (20 mL) and refluxed in the dark under nitrogen atmosphere overnight. The reaction mixture was cooled down to room temperature and evaporated in vacuo. The residue was purified by silica gel flash column chromatography (FCC, 3% diethyl ether and dichloromethane) and recrystallization from dichloromethane and hexane to give **H_2_-1** (26.7 mg, 37.0 μmol, 71%) as a black solid: UV-Vis (dichloromethane) λ_max_ 663 (rel., 0.48), 606 (0.08), 537 (0.09), 506 (0.09), 411 (1.00), 318 nm (0.20); ^1^H NMR (chloroform-*d*) δ 9.52 (1H, s, 10-H), 9.45 (1H, s, 5-H), 8.56 (1H, s, 20-H), 5.71 (2H, s, 3-CH_2_), 5.28, 5.12 (each 1H, d, *J* = 19 Hz, 13^1^-CH_2_), 4.50 (1H, dq, *J* = 2, 7 Hz, 18-H), 4.32 (1H, dt, *J* = 8, 2 Hz, 17-H), 3.99, 3.94 (each 1H, dt, *J* = 11, 7 Hz, 17^2^-COOCH_2_), 3.71 (2H, q, *J* = 8 Hz, 8-CH_2_), 3.68_3_ (3H, s, 12-CH_3_), 3.68_0_ (3H, s, 3^1^-OCH_3_), 3.41 (3H, s, 2-CH_3_), 3.27 (3H, s, 7-CH_3_), 2.74–2.64, 2.58–2.48, 2.37–2.20 (1H+1H+2H, m, 17-CH_2_CH_2_), 1.81 (3H, d, *J* = 7 Hz, 18-CH_3_), 1.70 (3H, t, *J* = 8 Hz, 8^1^-CH_3_), 1.51–1.40 (2H, m, 17^2^-COOCCH_2_), 1.29–1.03 (18H, m, 17^2^-COOC_2_(CH_2_)_9_), 0.85 (3H, t, *J* = 7 Hz, 17^2^-COOC_11_CH_3_), 0.40, –1.72 (each 1H, s, NH×2); HRMS (APCI) found: *m*/*z* 721.4683. Calcd. for C_45_H_61_N_4_O_4_: MH^+^, 721.4687.

Zinc 3-devinyl-methoxymethyl-pyropheophorbide-*a* dodecyl ester (**Zn-1**)

A methanol solution saturated with zinc acetate dihydrate (2 mL) was added to a dichloromethane solution (10 mL) of free-base **H_2_-1** (20.5 mg, 28.4 μmol) and stirred at room temperature in the dark under nitrogen atmosphere for 2 hours. The reaction mixture was washed with aqueous 4% sodium hydrogen carbonate and water, dried over sodium sulfate, and evaporated. The residue was washed with hexane and to give the corresponding zinc complex **Zn-1** (21.4 mg, 27.3 μmol, 96%) as a green solid: UV-Vis (tetrahydrofuran) λ_max_ 649 (rel., 0.75), 603 (0.09), 568 (0.05), 524 (0.03), 489 (0.01), 426 (1.00), 410 (0.53), 315 nm (0.18); ^1^H NMR (chloroform-*d*) δ 9.55 (1H, s, 10-H), 9.29 (1H, s, 5-H), 8.34 (1H, s, 20-H), 5.62, 5.59 (each 1H, d, *J* = 12 Hz, 3-CH_2_), 5.18, 5.06 (each 1H, d, *J* = 19 Hz, 13^1^-CH_2_), 4.39 (1H, dq, *J* = 2, 7 Hz, 18-H), 4.20 (1H, dt, *J* = 8, 2 Hz, 17-H), 3.97, 3.93 (each 1H, dt, *J* = 11, 7 Hz, 17^2^-COOCH_2_), 3.75 (2H, q, *J* = 8 Hz, 8-CH_2_), 3.69_2_ (3H, s, 3^1^-OCH_3_), 3.68_7_ (3H, s, 12-CH_3_), 3.33 (3H, s, 2-CH_3_), 3.26 (3H, s, 7-CH_3_), 2.59–2.49, 2.40–2.32, 2.31–2.22, 2.04–1.88 (each 1H, m, 17-CH_2_CH_2_), 1.71 (3H, d, *J* = 7 Hz, 18-CH_3_), 1.70 (3H, t, *J* = 8 Hz, 8^1^-CH_3_), 1.48 (2H, quintet, *J* = 7 Hz, 17^2^-COOCCH_2_), 1.26 (2H, sextet, *J* = 7 Hz, 17^2^-COOC_10_CH_2_), 1.23–1.15 (16H, m, 17^2^-COOC_2_(CH_2_)_8_), 0.85 (3H, t, *J* = 7 Hz, 17^2^-COOC_11_CH_3_); HRMS (APCI) found: *m*/*z* 783.3820. Calcd. for C_45_H_59_N_4_O_4_Zn: MH^+^, 783.3822.

3-Devinyl-3-methoxymethyl-pyropheophorbide-*a* *N*-[10-(*t*-butoxycarbamoyl)decyl] amide (**H_2_-6**)

Carboxylic acid **H_2_-5** (100.7 mg, 182.2 μmol) and BocNH(CH_2_)_10_NH_2_·HCl (66.9 mg, 217 μmol) were dissolved in distilled dichloromethane (20 mL). 1-Hydroxybenzotriazole (111.5 mg, 825.2 μmol), 1-ethyl-3-(3-dimethylaminopropyl)-carbodiimide hydrogen chloride (140.3 mg, 731.9 μmol), and triethylamine (115.0 mg, 1.136 mmol) were added to the solution and stirred at room temperature in the dark under nitrogen atmosphere. After stirring overnight, the reaction mixture was washed with aqueous 2% hydrochloric acid, aqueous 4% sodium hydrogen carbonate, and water, dried over sodium sulfate anhydrous, and evaporated. The residue was purified by FCC (1–2% methanol and dichloromethane) and recrystallization from dichloromethane and hexane to give amide **H_2_-5** (134.0 mg, 166.0 μmol, 91%) as a black solid: UV-Vis (dichloromethane) λ_max_ 663 (rel., 0.48), 607 (0.08), 537 (0.09), 506 (0.09), 411 (1.00), 318 nm (0.20); ^1^H NMR (chloroform-*d*) δ 9.45 (1H, s, 10-H), 9.43 (1H, s, 5-H), 8.55 (1H, s, 20-H), 5.69 (1H, s, 3-CH_2_), 5.25, 5.11 (each 1H, d, *J* = 19 Hz, 13^1^-CH_2_), 4.92 (1H, t, *J* = 6 Hz, 17^2^-CONH), 4.52 (1H, dq, *J* = 2, 8 Hz, 18-H), 4.45 (1H, br-t, *J* = 6 Hz, 17^2^-CONC_10_NH), 4.35 (1H, dt, *J* = 8, 2 Hz, 17-H), 3.68 (3H, s, 3^1^-OCH_3_), 3.68 (2H, q, *J* = 8 Hz, 8-CH_2_), 3.59 (3H, s, 12-CH_3_), 3.40 (3H, s, 2-CH_3_), 3.26 (3H, s, 7-CH_3_), 3.01 (2H, q, *J* = 7 Hz, 17^2^-CONC_9_CH_2_), 2.96, 2.88 (each 1H, ddt, *J* = 14, 6, 7 Hz, 17^2^-CONCH_2_), 2.73–2.62, 2.50–2.39, 2.23–2.13, 1.91–1.83 (each 1H, m, 17-CH_2_CH_2_), 1.80 (3H, d, *J* = 8 Hz, 18-CH_3_), 1.68 (3H, t, *J* = 8 Hz, 8^1^-CH_3_), 1.41 (9H, s, 17^2^-CONC_10_NCOOC(CH_3_)_3_), 1.36 (2H, quintet, *J* = 7 Hz, 17^2^-CONC_8_CH_2_), 1.22–0.93 (14H, m, 17^2^-CONC(CH_2_)_7_), 0.39, –1.72 (each 1H, s, NH×2); HRMS (APCI) found: *m*/*z* 807.5171. Calcd. for C_48_H_67_N_6_O_5_: MH^+^, 807.5167.

3-Devinyl-3-methoxymethyl-pyropheophorbide-*a* *N*-[10-(3-butylureido)decyl] amide (**H_2_-2**)

To a solution of Boc-protected amine **H_2_-6** (30.5 mg, 37.8 μmol) was added 4 N hydrochloric acid in ethyl acetate (10 mL) and stirred at room temperature in the dark for 2 hours. The reaction mixture was evaporated. The residue was dissolved in dry dichloromethane solution (10 mL) of *n*-butylisocyanate (3.8 mg, 38.3 μmol) and triethylamine (3.8 mg, 37.5 μmol) and stirred at room temperature in the dark under nitrogen atmosphere for 2 hours. The reaction mixture was washed with aqueous 2% hydrochloric acid, aqueous 4% sodium hydrogen carbonate, and water, dried over sodium sulfate, and evaporated. The residue was purified by silica gel open column chromatography (3% methanol and dichloromethane) and recrystallization from dichloromethane containing a small amount of methanol and hexane to give urea **H_2_-2** (17.0 mg, 21.1 μmol, 56%) as a black solid: UV-Vis (chloroform) λ_max_ 664 (rel., 0.51), 608 (0.08), 538 (0.10), 507 (0.10), 412 (1.00), 320 nm (0.21); ^1^H NMR (10% methanol-*d*_4_ and chloroform-*d*) δ 9.49 (1H, s, 10-H), 9.40 (1H, s, 5-H), 8.56 (1H, s, 20-H), 6.08 (1H, br-s, 17^2^-CONH), 5.70 (2H, s, 3-CH_2_), 5.26, 5.11 (each 1H, d, *J* = 19 Hz, 13^1^-CH_2_), 4.90 (2H, br-s, 17^2^-CONC_10_NHCONH), 4.53 (1H, dq, *J* = 2, 7 Hz, 18-H), 4.31 (1H, dt, *J* = 7, 2 Hz, 17-H), 3.70_5_ (3H, s, 3^1^-OCH_3_), 3.69_7_ (2H, q, *J* = 8 Hz, 8-CH_2_), 3.64 (3H, s, 12-CH_3_), 3.40 (3H, s, 2-CH_3_), 3.26 (3H, s, 7-CH_3_), 3.06 (2H, t, *J* = 7 Hz, 17^2^-CONC_10_NCONCH_2_), 3.01 (2H, t, *J* = 7 Hz, 17^2^-CONC_9_CH_2_), 3.00–2.89 (2H, m, 17^2^-CONCH_2_), 2.71–2.61, 2.43–2.34, 2.31–2.23, 2.04–1.95 (each 1H, m, 17-CH_2_CH_2_), 1.81 (3H, d, *J* = 8 Hz, 18-CH_3_), 1.70 (3H, t, *J* = 8 Hz, 8^1^-CH_3_), 1.41 (2H, quintet, *J* = 7 Hz, 17^2^-CONC_10_NCONCCH_2_), 1.36 (2H, br-quintet, *J* = 7 Hz, 17^2^-CONC_8_CH_2_), 1.19 (2H, sextet, *J* = 7 Hz, 17^2^-CONC_10_NCONC_2_CH_2_), 1.24–1.02 (14H, m, 17^2^-CONC(CH_2_)_7_), 0.89 (3H, t, *J* = 7 Hz, 17^2^-CONC_10_NCONC_3_CH_3_), 0.55, –1.57 (each 1H, s, NH×2); HRMS (APCI) found: *m*/*z* 806.5327. Calcd. for C_48_H_68_N_7_O_4_: MH^+^, 806.5327.

Zinc 3-devinyl-3-methoxymethyl-pyropheophorbide-*a* *N*-[10-(3-butylureido)decyl] amide (**Zn-2**)

Similar to the synthesis of **Zn-1**, zinc metallation of free-base **H_2_-2** (10.0 mg, 12.4 μmol) and successive recrystallization from dichloromethane and hexane gave the corresponding zinc complex **Zn-2** (9.8 mg, 11.3 μmol, 91%) as a dark green solid: UV-Vis (tetrahydrofuran) λ_max_ 649 (rel., 0.73), 604 (0.10), 568 (0.06), 522 (0.04), 489 (0.03), 426 (1.00), 405 (0.55), 315 nm (0.21); ^1^H NMR (1% pyridine-*d*_5_ and chloroform-*d*) δ 9.55 (1H, s, 10-H), 9.29 (1H, s, 5-H), 8.34 (1H, s, 20-H), 5.60 (2H, s, 3-CH_2_), 5.21 (1H, t, *J* = 6 Hz, 17^2^-CONH), 5.14, 5.06 (each 1H, d, *J* = 19 Hz, 13^1^-CH_2_), 4.42 (1H, dq, *J* = 2, 7 Hz, 18-H), 4.22 (1H, dt, *J* = 7, 2 Hz, 17-H), 3.94 (2H, t, *J* = 6 Hz, 17^2^-CONC_10_NHCONH), 3.75 (2H, q, *J* = 8 Hz, 8-CH_2_), 3.70 (3H, s, 3^1^-OCH_3_), 3.66 (3H, s, 12-CH_3_), 3.32 (3H, s, 2-CH_3_), 3.26 (3H, s, 7-CH_3_), 3.00–2.80 (6H, m, 17^2^-CONCH_2_C_8_CH_2_NCONCH_2_), 2.60–2.50, 2.39–2.28, 2.07–1.97, 1.62–1.55 (each 1H, m, 17-CH_2_CH_2_), 1.70_3_ (3H, d, *J* = 7 Hz, 18-CH_3_), 1.70_0_ (3H, t, *J* = 8 Hz, 8^1^-CH_3_), 1.33–0.99 (20H, m, 17^2^-CONC(CH_2_)_8_CNCONC(CH_2_)_2_), 0.84 (3H, t, *J* = 7 Hz, 17^2^-CONC_10_NCONC_3_CH_3_); HRMS (APCI) found: *m*/*z* 868.4465. Calcd. for C_48_H_66_N_7_O_4_Zn: MH^+^, 868.4462.

**3. Properties of zinc 3^1^-methoxy-chlorophyll derivatives**


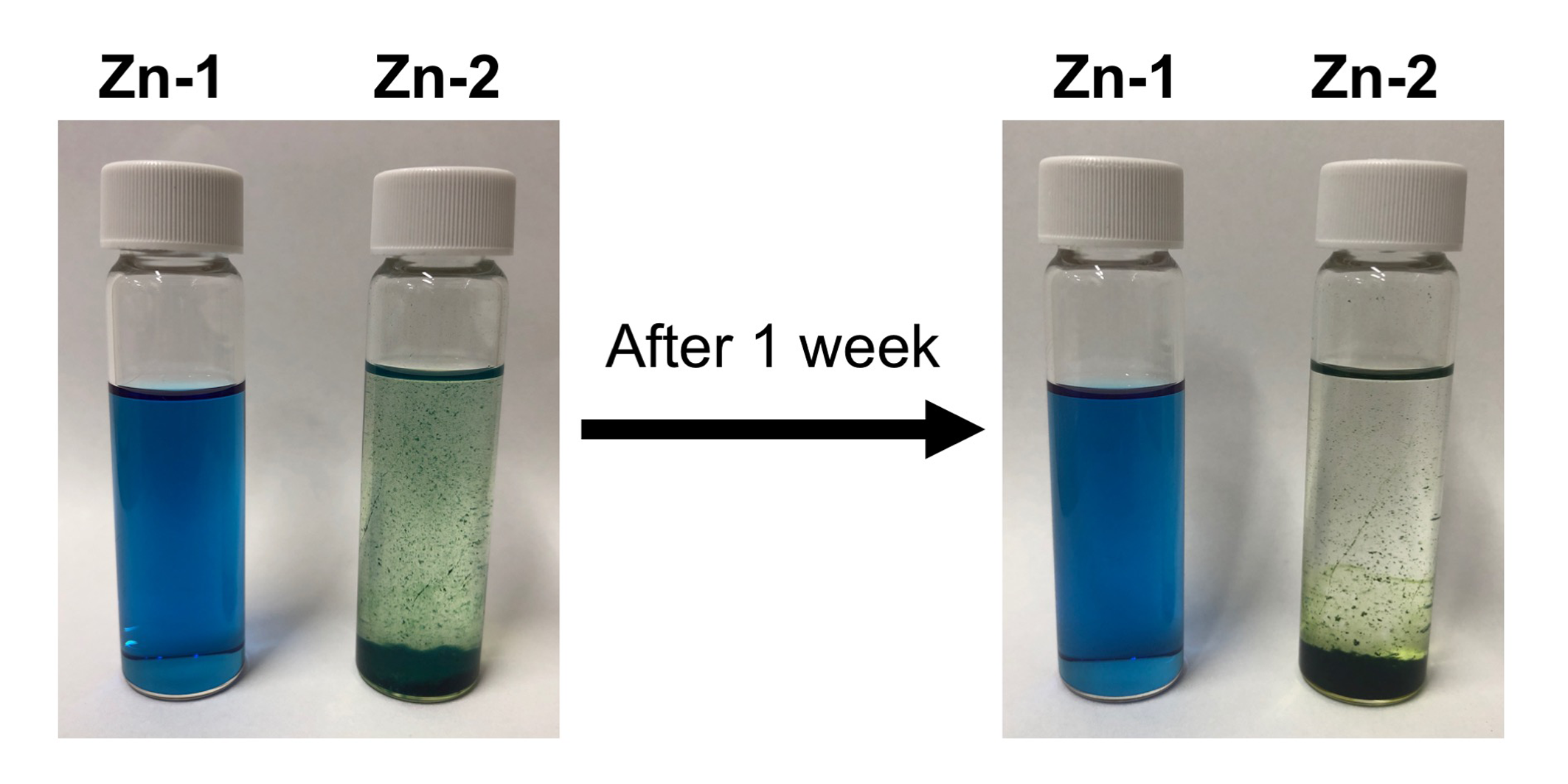


**Figure S1.** Photographs of **Zn-1** and **Zn-2** samples (100 μM) in THF/hexane (5:95, vol/vol) just after preparation (left) and after standing for 1 week (right).

**
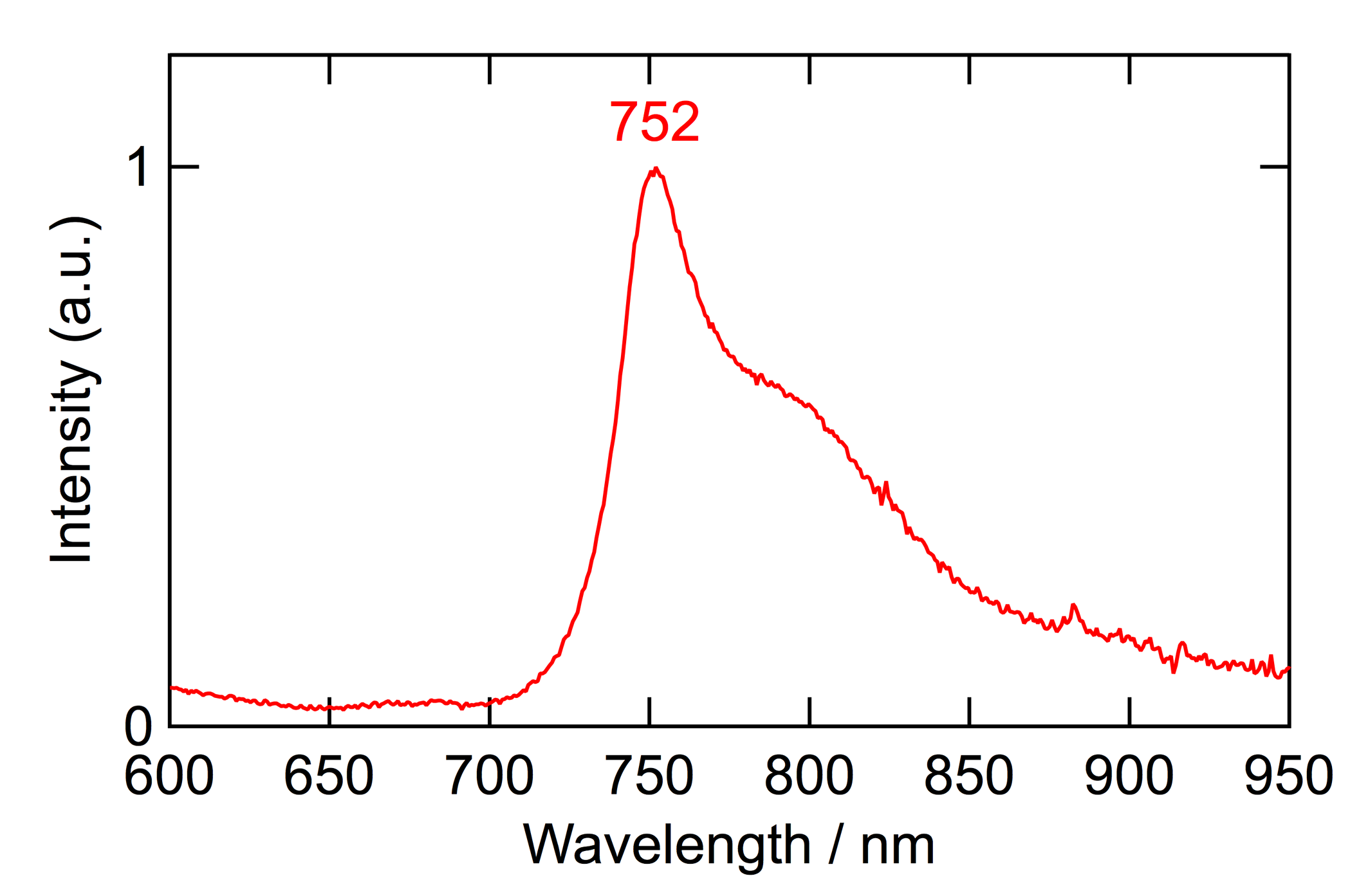
**

**Figure S2.** Fluorescence emission spectrum of **Zn-2** *J*-aggregates (after standing for one week) on a quartz substrate at the excitation of Soret band, λ_ex_ = 440 nm.


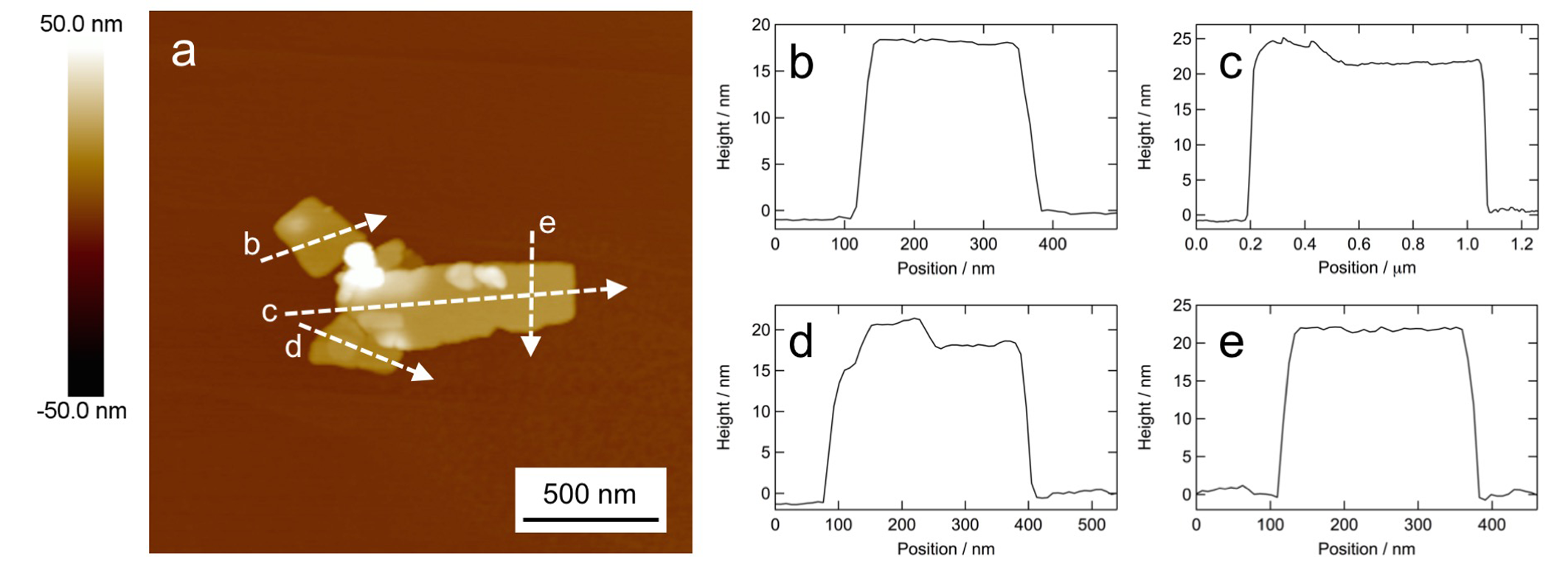


**Figure S3.** Tapping mode AFM analysis of **Zn-2** solids (after standing for one week) on an HOPG substrate, **a** height image and **b**–**e** cross-section analysis along white arrows in the image **a**.


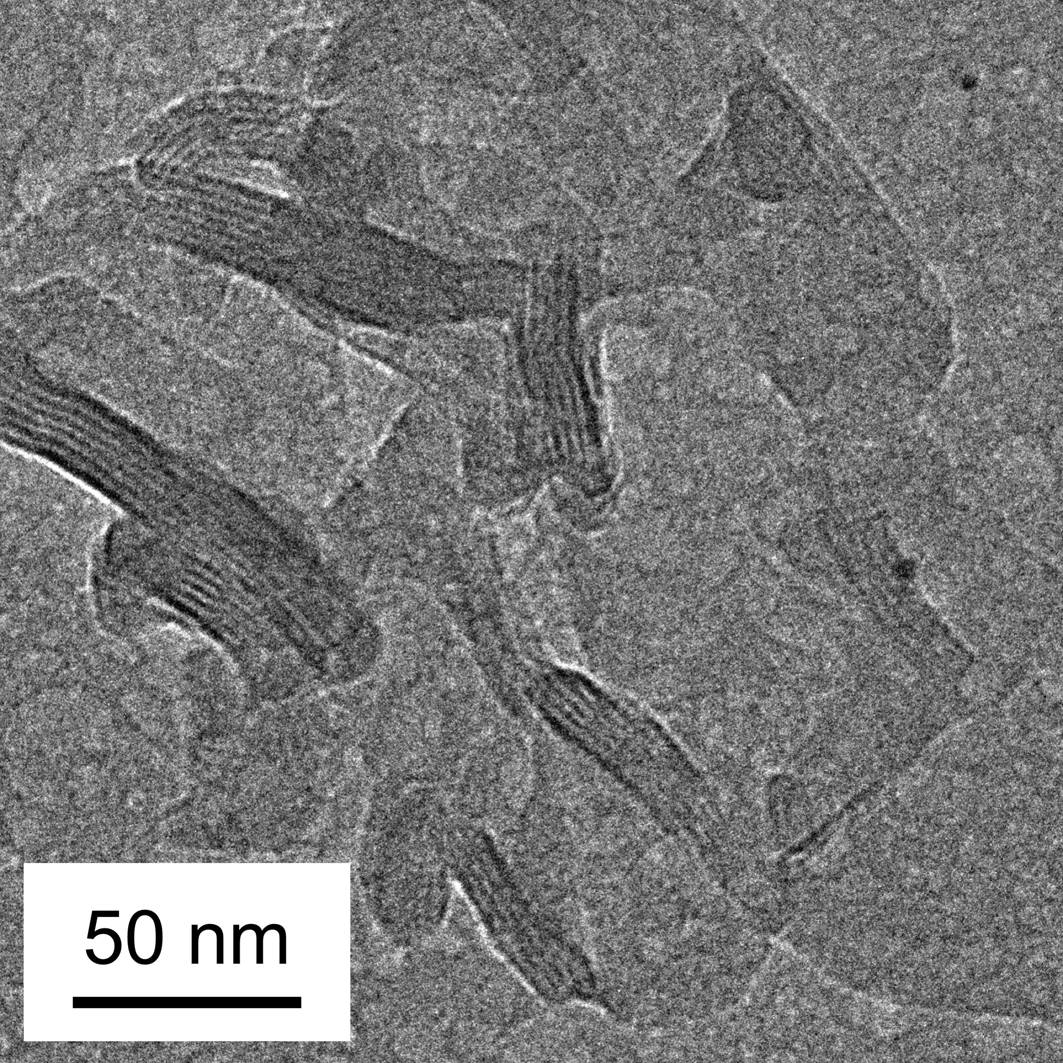


**Figure S4.** A cryo-TEM image of partially formed rod-shaped supramolecular nanostructure of **Zn-2** *J*-aggregates.


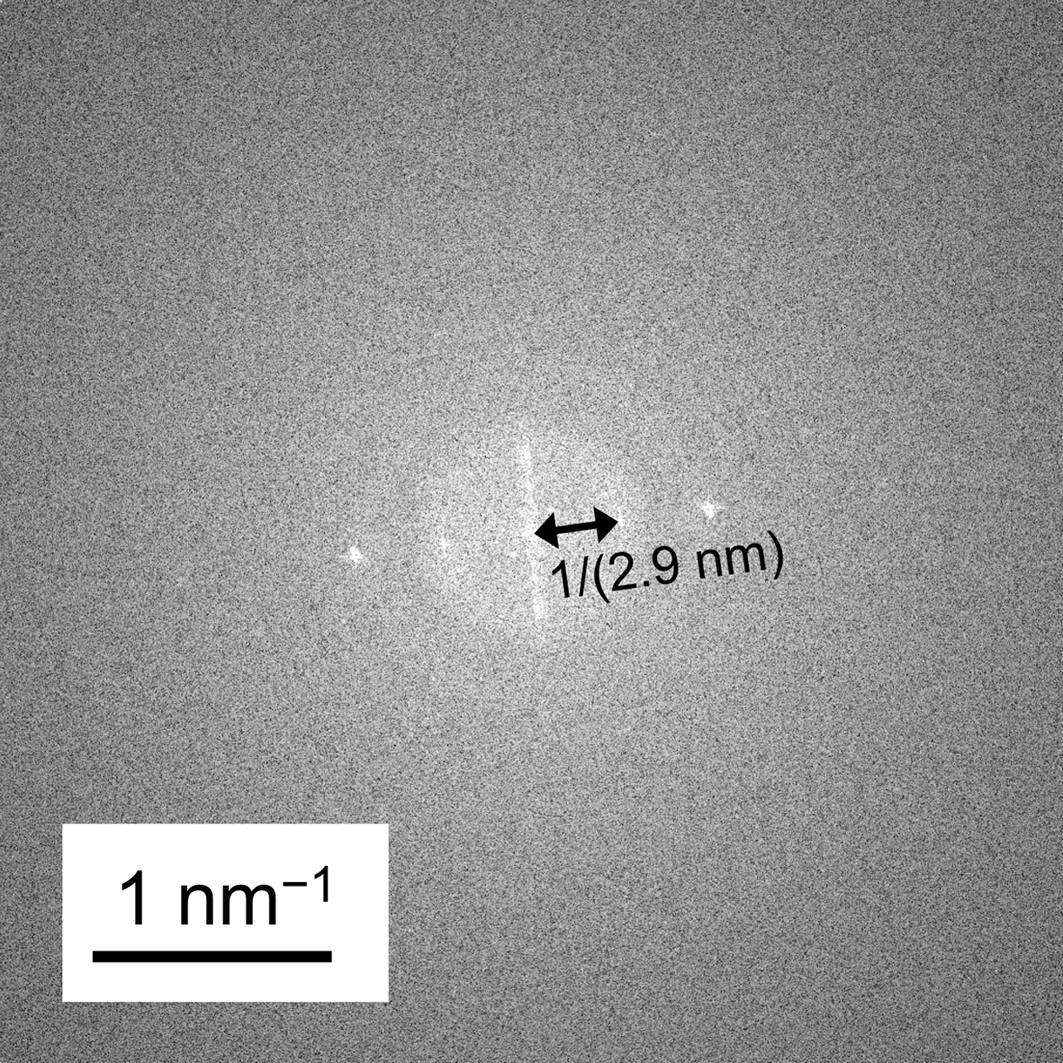


**Figure S5.** Fast Fourier transformation of the Figure 3d image in the main text.

**4. Supplementary references**

1. Tamiaki, H., Amakawa, M., Shimono, Y., Tanikaga, R., Holzwarth, A. R. & Schaffner, K. Synthetic zinc and magnesium chlorin aggregates as models for supramolecular antenna complexes in chlorosomes of green photosynthetic bacteria. *Photochem. Photobiol.* **63**, 92–99 (1996).
2. Miyatake, T., Tanigawa, S., Kato, S. & Tamiaki, H. Aqueous self-aggregates of amphiphilic zinc 3^1^-hydroxy- and zinc 3^1^-methoxy-chlorins for supramolecular light-harvesting systems. *Tetrahedron Lett.* **48**, 2251–2254 (2007).
3. Huber, V., Lysetska, M. & Würthner, F. Self-assembled single- and double-stack π-aggregates of chlorophyll derivatives on highly ordered pyrolytic graphite, *Small*, **3**, 1007–1014 (2007).
4. Ng, K. K., Takada, M., Harmatys, K., Chen, J. & Zheng, G. Chlorosome-inspired synthesis of templated metallochlorin-lipid nanoassemblies for biomedical application, *ACS Nano*, **10**, 4092–4101 (2016).
